# Structural analysis of the housecleaning nucleoside triphosphate pyrophosphohydrolase MazG from *Mycobacterium tuberculosis*

**DOI:** 10.3389/fmicb.2023.1137279

**Published:** 2023-03-01

**Authors:** Sen Wang, Baocai Gao, Anke Chen, Zhifei Zhang, Sheng Wang, Liangdong Lv, Guoping Zhao, Jixi Li

**Affiliations:** ^1^State Key Laboratory of Genetic Engineering, School of Life Sciences and Huashan Hospital, MOE Engineering Research Center of Gene Technology, Shanghai Engineering Research Center of Industrial Microorganisms, Fudan University, Shanghai, China; ^2^Shanghai Zelixir Biotech Company Ltd., Shanghai, China; ^3^School of Basic Medical Sciences, Fudan University, Shanghai, China; ^4^Key Laboratory of Synthetic Biology, CAS Center for Excellence in Molecular Plant Sciences, Shanghai Institute of Plant Physiology and Ecology, Chinese Academy of Sciences, Shanghai, China; ^5^Shanghai Key Laboratory of Infectious Diseases and Biosafety Emergency Response, National Medical Center for Infectious Diseases, Huashan Hospital, Fudan University, Shanghai, China

**Keywords:** *Mycobacterium tuberculosis*, MazG, NTP pyrophosphatases, crystal structure, SAXS

## Abstract

The housecleaning enzyme of *Mycobacterium tuberculosis* (Mtb), MazG, is a nucleoside triphosphate pyrophosphohydrolase (NTP-PPase) and can hydrolyze all canonical or non-canonical NTPs into NMPs and pyrophosphate. The *Mycobacterium tuberculosis* MazG (Mtb-MazG) contributes to antibiotic resistance in response to oxidative or nitrosative stress under dormancy, making it a promising target for treating TB in latent infection patients. However, the structural basis of Mtb-MazG is not clear. Here we describe the crystal structure of Mtb-MazG (1–185) at 2.7 Å resolution, composed of two similar folded spherical domains in tandem. Unlike other all-α NTP pyrophosphatases, Mtb-MazG has an N-terminal extra region composed of three α-helices and five β-strands. The second domain is global, with five α-helices located in the N-terminal domain. Gel-filtration assay and SAXS analysis show that Mtb-MazG forms an enzyme-active dimer in solution. In addition, the metal ion Mg^2+^ is bound with four negative-charged residues Glu119, Glu122, Glu138, and Asp141. Different truncations and site-directed mutagenesis revealed that the full-length dimeric form and the metal ion Mg^2+^ are indispensable for the catalytic activity of Mtb-MazG. Thus, our work provides new insights into understanding the molecular basis of Mtb*-*MazG.

## Introduction

Tuberculosis (TB), caused by *Mycobacterium tuberculosis* (Mtb), is one of the infectious killers globally that accounts for about 1.4 million deaths worldwide each year (Dartois and Rubin, 2022; Yang et al., 2022). Introducing the *Mycobacterium* bovis bacille Calmette-Guérin (BCG) vaccine in newborn babies has dramatically decreased the threat of Mtb (Lange et al., 2022). However, BCG is less effective for preventing pulmonary tuberculosis in adults and may have side effects in the immunocompromised hosts (Furin et al., 2019). In addition, Mtb can evade the host immune system in a dormant way in latently infected patients, making the antibacterial drug development challenging (de Wet et al., 2019). Therefore, it is particularly important to understand the molecular events of growth control and metabolic adaptation of non-growing Mtb for developing new therapeutic strategies.

Cellular metabolism is precisely regulated by various housecleaning enzymes, especially the NTP pyrophosphatases, which hydrolyze wasted compounds into cellular metabolites, therefore preventing the non-canonical NTPs-triggered mutagenesis and DNA damage (Gad et al., 2014; Fan et al., 2018). Housecleaning NTP pyrophosphatases include four structural superfamilies: trimeric dUTPase, ITPase (Maf/HAM1), Nudix-box containing hydrolases, and all-α NTP pyrophosphatases (Galperin et al., 2006). In addition, structure-based analysis reveals that the all-α NTP pyrophosphatases include the dimeric dUTPase, the phosphoribosyl-ATP pyrophosphatase HisE, and the NTP pyrophosphatase MazG (Moroz et al., 2005). All the enzymes specifically target non-canonical NTPs, including 5-OH-dCTP, dUTP, dITP, 2-oxo-dATP, and 8-oxo-dGTP, with high affinities (Galperin et al., 2006; Lu et al., 2010; Lyu et al., 2013).

MazG, identified initially as a downstream gene of the toxin-antitoxin complex MazEF in *E. coli*, exists in different bacteria and many phages (Zhang and Inouye, 2002; Huang et al., 2021). Previous studies showed that Mtb-MazG is able to hydrolyze all canonical (d)NTPs and 8-oxo-dGTP (Lu et al., 2010). Furthermore, MazG eliminates 5-OH-dCTP and regulates pyrimidine metabolism, safeguarding the genetic stability of Mtb during oxidative stress conditions (Lyu et al., 2013). On the other way, MazG is required for the persistence of Mtb during chronic infection of mice and contributes to antibiotic tolerance of stationary-phase culture and intracellular Mtb (Shi et al., 2019). Currently, the structures of MazG from *Bacillus anthracis*, *E. coli*, and *Deinococcus radiodurans* reveal a quite similar dimeric or tetrameric all-α-helical architecture (Lee et al., 2008; Goncalves et al., 2011; Kim and Hong, 2016). However, Mtb-MazG shows not only a certain extent of sequence similarity with bacterial homologs, but also has one extra region at the N-terminal domain, which may contribute to the antibacterial resistance in the dormant stage.

Here, we report the crystal structure of MazG (1–185) from *M. tuberculosis* at 2.7 Å resolution, forming a dimer through the interaction of two repeated MazG-like domains. Structural alignment and mutation studies revealed that the magnesium-ion-binding sites and full-length dimeric protein are necessary for the catalytic activity of MazG. The structural basis of MazG might provide insights into understanding the diverse functions of MazG in dormant Mtb.

## Results

### The purification and crystallization of Mtb*-*MazG

**Figure 1:**
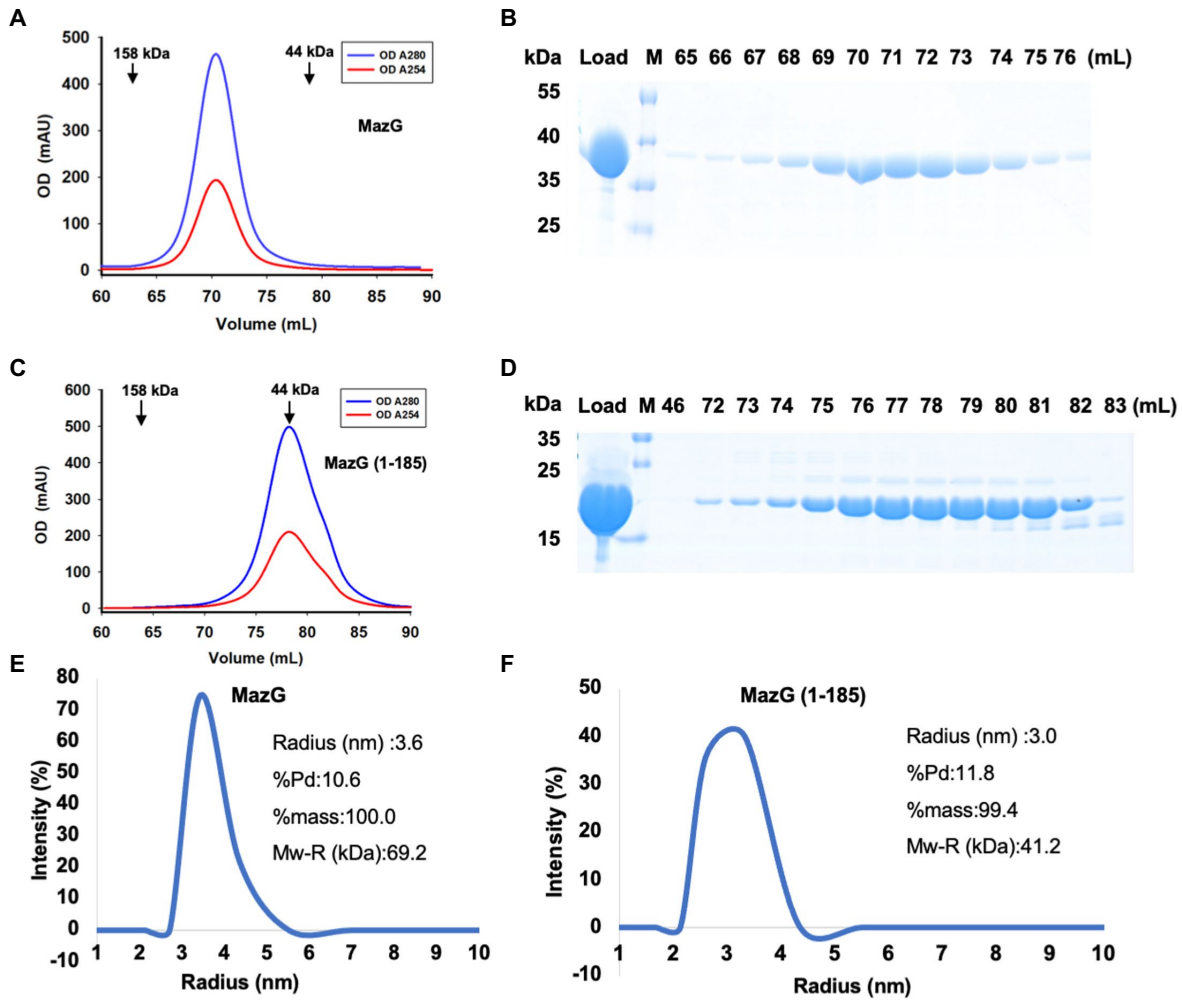
Characterization of Mtb-MazG and mutant (1–185) by gel-filtration and DLS methods. **(A,C)** Gel-filtration profiles of MazG and MazG (1–185) on a Superdex 200 16/600 column. **(B,D)** SDS-PAGE of the corresponding elution fractions in **A,C**. M: protein markers. **(E,F)** The DLS distributions of MazG and MazG (1–185).

To reveal the structure of Mtb-MazG, we expressed the 6xHis-MazG fusion protein in *Escherichia coli* BL21(DE3) cells and further purified with different chromatographies as previously described (Zhan et al., 2022). The purified Mtb-MazG came out at the peak of ~71 mL on a Superdex200 16/600 column, corresponding with a molecular weight of ~75 kD. As the theoretical molecular weight of Mtb-MazG is 35 kD, it showed that MazG was a dimer in solution (Figures 1A,B). Next, we screened more than 1,000 crystallizing conditions for the high purity (>95%) full-length Mtb-MazG protein. However, no crystals were grown, which probably resulted from the intrinsic disorder property (Chen et al., 2022). Thus, to identify the suitable regions for crystallization, we performed limited protease digestion for Mtb-MazG. The results showed that Mtb-MazG was cleaved into two stable fragments by endoproteinase Glu-C (Supplementary Figure S1A). Further mass spectrometry (MS) experiment showed that the upper band and lower band in SDS-PAGE might be a partial fragment of the N-terminal part (1–185) and the C-terminal part (186–325) of Mtb-MazG, respectively (Supplementary Figures S1A,B). To check whether the two parts interacted with each other, we performed the gel-filtration analysis with the endoproteinase-digested Mtb-MazG protein *in vitro*. However, endoproteinase digestion resulted in more than two fragments less than 15 kDa, and this could have hindered the formation of a stable complex of the two parts of MazG (Supplementary Figures S1C,D). In addition, we constructed a series of truncated versions, including MazG (1–185), MazG (1–231), MazG (1–281), MazG (1–305), MazG (186–305), MazG (186–325), and MazG (85–325) for crystallization. Specifically, MazG (1–185) came out at the peak of ~78 mL on the Superdex200 16/600 column, corresponding with a molecular weight of ~40 kD. Therefore, MazG (1–185) was a dimer in solution, as the theoretical molecular weight of MazG (1–185) is 20 kD (Figures 1C,D). Also, the dynamic light scattering (DLS) experiment showed that the radius and estimated molecular weight of full-length Mtb-MazG and Mtb-MazG (1–185) were 3.6 Å and 3.0 Å, 69.2 kD and 41.2 kD, respectively, indicating both of they possessed good homogeneities in solution (Figures 1E,F).

### The overall structure of MazG (1–185)

**Figure 2:**
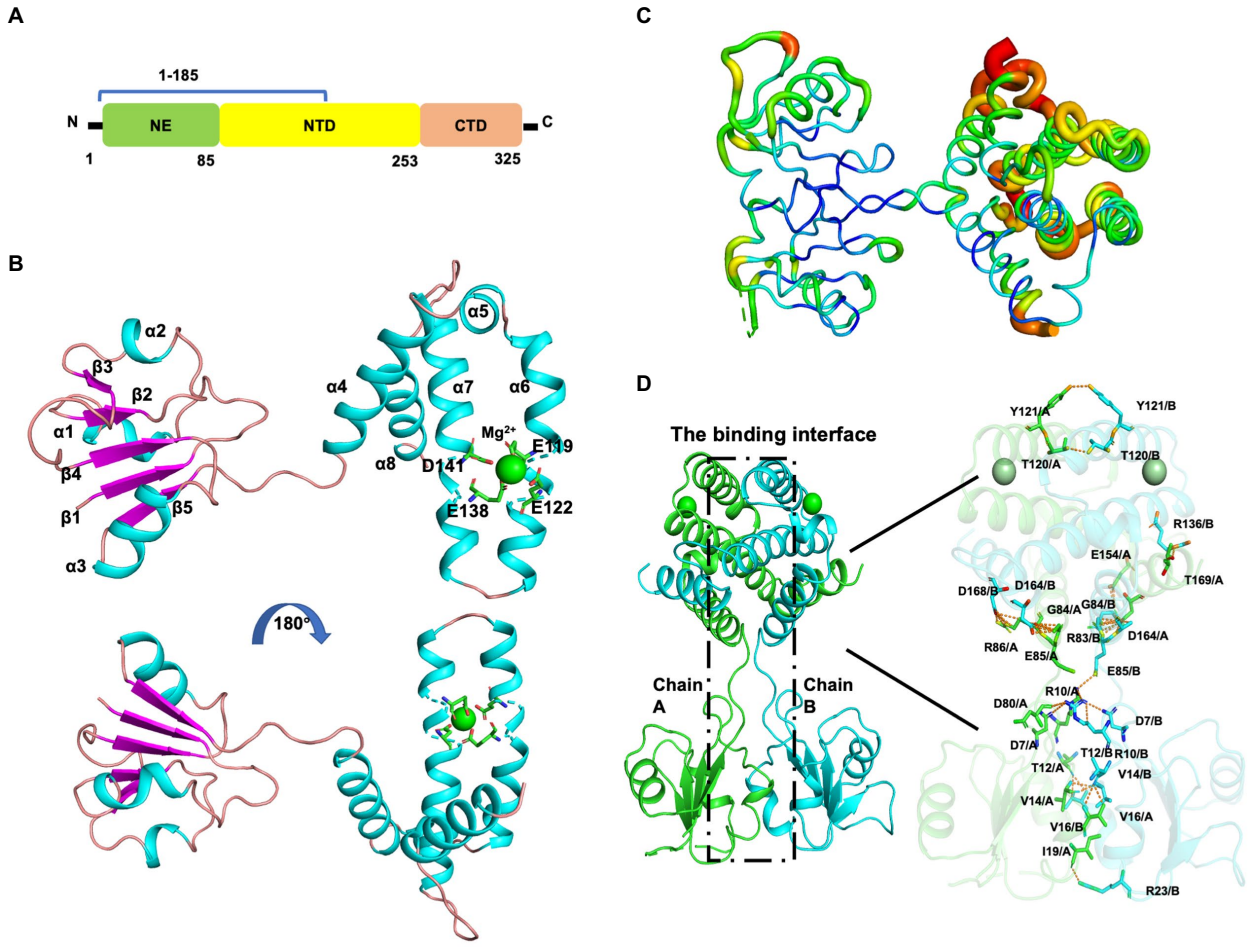
The overall structure of MazG (1–185) from *Mycobacterium tuberculosis*. **(A)** Schematic of the Mtb-MazG protein. Mtb-MazG is composed of the N-terminal domain (NTD), the C-terminal domain (CTD), and the N-terminal extra region (NE). **(B)** The crystal structure of Mtb-MazG (1–185) is represented by a cartoon model, which is composed of eight α-helices and five β-strand. The magnesium is bound with four residues that are located on α6 and α7 of MazG (1–185). Cyan: α-helix; magenta: β-strand; green: magnesium ion. **(C)** The B-factor distribution of Mtb-MazG protein. The wider and redder tubing indicated a higher B-factor. **(D)** The binding interface between the swapped-dimeric Mtb-MazG (1–185). The dashed black box (left) denotes the interface between the swapped dimeric structure. The key residues (right) involved in the interface are shown in stick models. Magnesium ions are shown in green spheres.

Mtb*-*MazG mainly contains three domains, an extra N-terminal region (NE, residues 1–85) that is absent in other bacterial homologs, the N-terminal domain (NTD, residues 86–253), and the C-terminal domain (CTD, residues 254–325; Figure 2A; Mota et al., 2016). Despite failing in the crystallization of full-length MazG, we succeeded in obtaining the MazG (1–185) protein crystals and solved the 2.7 Å resolution structure using molecular replacement of the AlphaFold2-predicted structure (AF-P96379-F1). There are six MazG molecules in an asymmetrical unit with a cell dimension of 109 × 109 × 242 Å, which includes two monomers and two dimers consisting of two identical chains. The Mtb-MazG (1–185) monomer contained eight α-helices and five β-stands, comprising two globular domains, including a primary three-layered α/β/α sandwich domain (named domain 1) and an entirely α-helices-composed domain (named domain 2; Figure 2B). Domain 1 was composed of three α-helices (α1, α2, and α3) and five β-sheet strands (β1, β2, β3, β4, and β5), which was absent in other NTP pyrophosphatases, including *B. anthracis*, *E. coli*, and *D. radiodurans* MazGs (Lee et al., 2008; Goncalves et al., 2011; Kim and Hong, 2016). Domain 2 included five α-helices (α4, α5, α6, α7, and α8), which showed a higher B-factor than that in domain 1, indicating this region exhibited more flexibility (Figure 2C). The two domains were connected with a short linker (Ala78-Gly84). Helices α3, α4, α5, α6, and α7 flanked one side of the β-sheet, while α1, α2, and α8 were located on the opposite side (Figure 2B). Also, Mtb-MazG (1–185) forms a dimer in crystal packing, with a stable conformation in the interaction region (Figure 2C). The dimeric Mtb-MazG (1–185) is with an approximate dimension of 45 × 47 × 60 Å (Figure 2D). The interface-involved residues of the swapped-dimeric MazG were located in α1 of domain 1, and α4, α6, and α8 of domain 2, of which fifteen residues formed multiple hydrogen bonds and salt bridges in relative distances within 3.5 Å (Figure 2D). These residues included D7, R10, T12, V14, V16, I19, R23, G84, E85, R86, T120, Y121, R136, E154, D164, D168, and T169 (Figure 2D).

**Figure 3:**
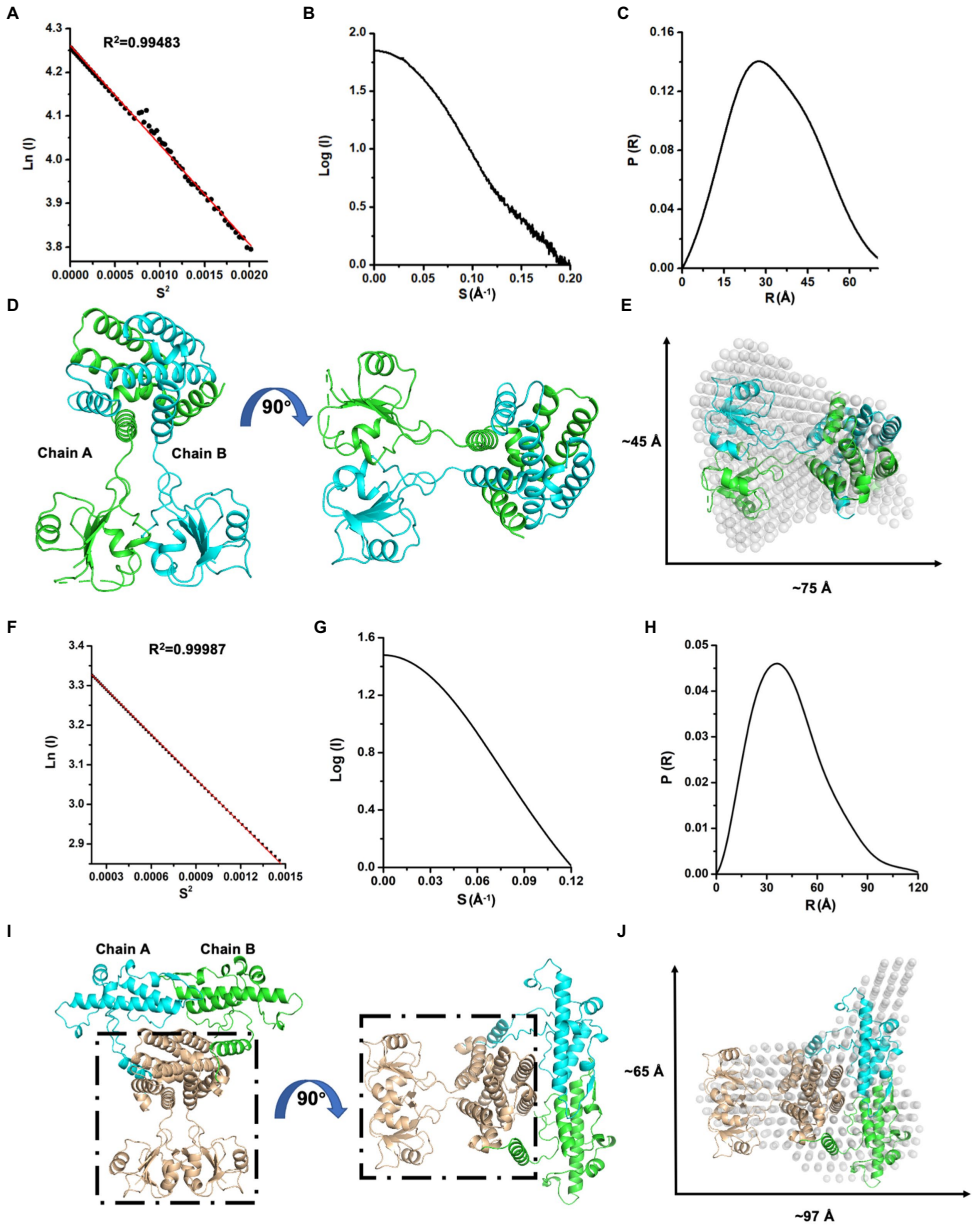
SAXS analysis of Mtb-MazG and its truncation MazG (1–185). **(A–C)** Guinier plot, intensity profile, and the P(R) curve of MazG (1–185). **(D)** The dimeric structure of MazG (1–185). The two subunits are colored cyan and green, respectively. **(E)** The de-novo *DAMMIN* model was overlapped with the crystal structure of MazG (1–185; PDB code: 7YH5, grey). **(F–H)** Guinier plot, intensity profile, and the P(R) curve of full-length Mtb-MazG. **(I)** The AlphaFold2 predicted-dimeric structure of full-length MazG. Chains A and B are shown in cyan and green, respectively. The region (1–185) is shown in wheat. **(J)** The de-novo *DAMMIN* model was overlapped with the AlphaFold2-predicted structure of full-length MazG (AF-P96379-F1).

Next, we investigated the solution status of MazG (1–185) and full-length MazG by the small-angle X-ray scattering (SAXS) method (Figure 3). MazG (1–185) and full-length MazG behaved well in solution, evidenced by the Guinier plots and intensity profiles (Figures 3A,B,F,G). The maximum dimensions (Dmax) from the distance distribution function p(r) of MazG (1–185) and full-length MazG were around 68 Å and 120 Å, respectively (Figures 3C,H). Moreover, when superimposed the crystal structure of MazG (1–185) with the *ab initio* envelope obtained from the *de-novo* DAMMIN model of SAXS, MazG (1–185) showed high similarities, confirming that the purified MazG (1–185) *in vitro* was indeed the active dimeric form (Figures 3D,E). For full-length MazG, we constructed the dimeric structure from the AlphaFold2 model (AF-P96379-F1; Figure 3I). Also, the dimeric structure overlapped well with the *ab initio* envelope obtained from the *de-novo* DAMMIN model of SAXS (Figure 3J).

### The magnesium-binding sites and enzymatic activity

**Figure 4:**
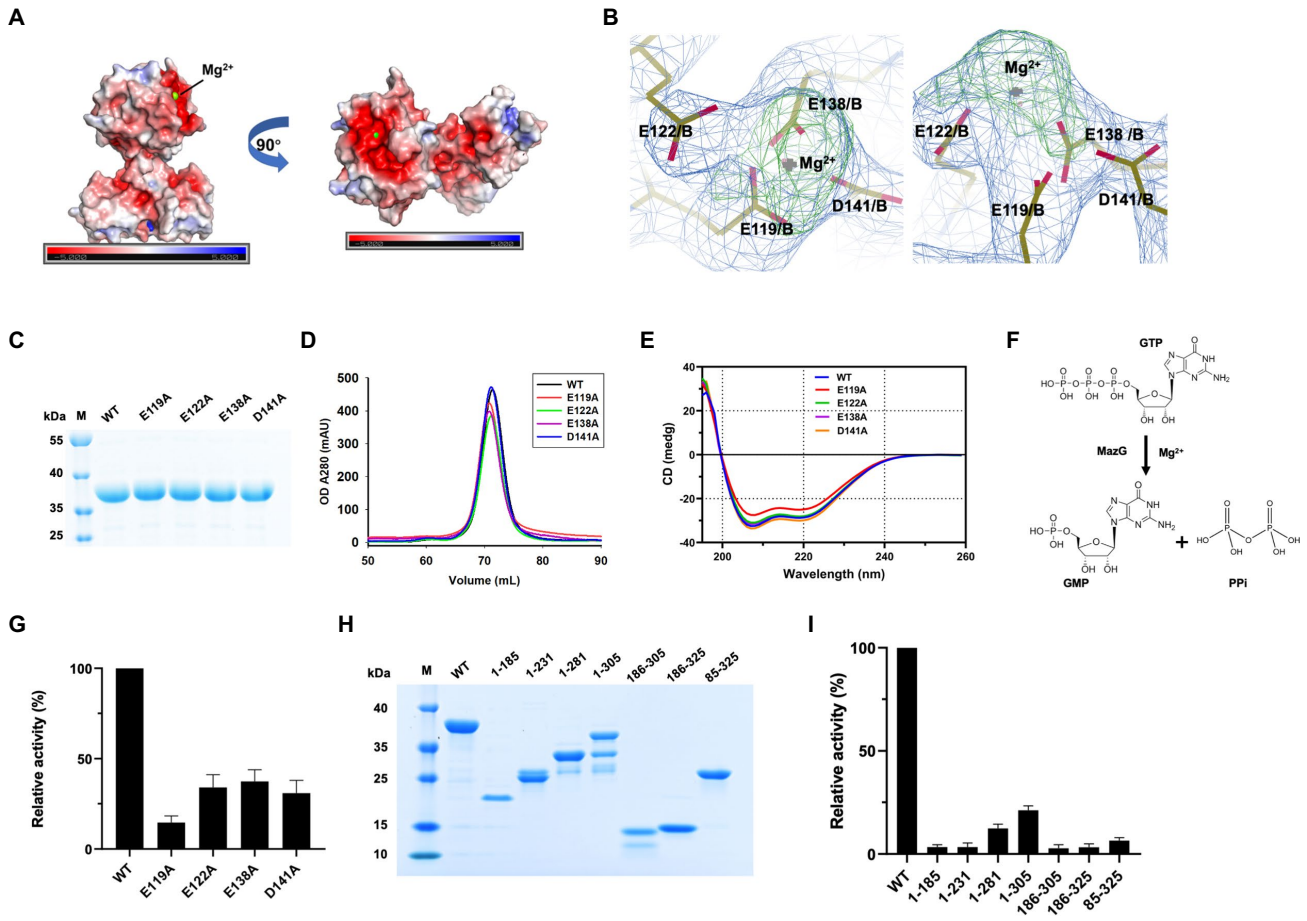
The magnesium binding sites and enzymatic characterizations of Mtb-MazG. **(A)** The surface potential charge of MazG (1–185) structure. Red: negative charge; blue: positive charge. **(B)** The electron density map (2Fo-Fc, level = 1.0) of magnesium-bound residues in MazG. **(C)** SDS-PAGE of MazG and different mutants. **(D)** The gel filtration profiles of MazG and different mutants. **(E)** CD spectra of MazG and its mutants measured at 18°C. Each spectrum reported here is an average of three scans. **(F)** Schematic diagram of the enzymatic reaction. MazG hydrolyzes GTP into GMP and pyrophosphoric acid. **(G)** The relative activities of WT and different mutants of Mtb-MazG. The activity of WT was taken as 100%. **(H)** SDS-PAGE of WT MazG and its various truncations. Notably, MazG (1–281), MazG (1–305), and MazG (186–305) showed degradation during the purification process. **(I)** The relative activities of WT MazG and different truncations. The activity of WT was taken as 100%.

The two-metal-ion mechanism is conserved in the MazG family across bacteria to phages (Mota et al., 2016; Huang et al., 2021; Wood et al., 2021). In the MazG (1–185) structure, one magnesium ion was present per subunit. The Mg^2+^ was coordinated by three glutamate residues (Glu119, Glu122, and Glu138) and one aspartate (Asp141; Figure 2B). Surface charge and electron density map analysis showed that Mg^2+^ was surrounded by negative charges, which are conserved in the NTP pyrophosphatase superfamily (Figures 4A,B). The four residues were mutated into alanines to unveil further the critical roles for enzymatic activity (Figure 4C). Compared with the wild-type (WT) MazG protein, the mutants came out at the same peak on gel-filtration profiles, indicating they were also dimer in solution (Figure 4D). Also, given that mutants were essential for catalysis, to exclude the possible reason that mutations affect the enzyme activity by changing the original structural conformation of the protein, we performed circular dichroism spectra (CD) analysis with the WT MazG and mutant proteins. The CD results showed that the four mutants did not change the structural conformation of the MazG (Figure 4E).

The hydrolase activity of Mtb-MazG was assayed with a spectrophotometric method using GTP as the substrate, as previously described (Lyu et al., 2013). The wild type Mtb-MazG possessed a *kcat* value of (0.83 ± 0.06) s^−1^ and a *K*m value of (0.4 ± 0.1) mM, respectively. The catalytic efficiency (*kcat*/*K*m) of the MazG protein toward GTP was 2.1 mM^−1^ s^−1^. Also, the NTP-PPase activity was significantly lowered for the mutants of E119A, E122A, E138A, and D141A (Figures 4F,G). Among the four mutants, E119A has the lowest enzymatic activity, whereas E122A, E138A, and D141A possess 20 ~ 40% relative activities with that in WT MazG. Moreover, the enzyme activities toward different truncations (1–185, 1–231, 1–281, 1–305, 186–305, 186–325, and 85–325) were measured and found to be significantly lower in all cases compared to WT MazG (Figures 4H,I), which showed similar enzyme activity to our previous reported (Lyu et al., 2013). Taken together, the metal ion and full-length protein are indispensable for the activity of MazG in Mtb.

### Structural comparison of Mtb-MazG with its homologs

**Figure 5:**
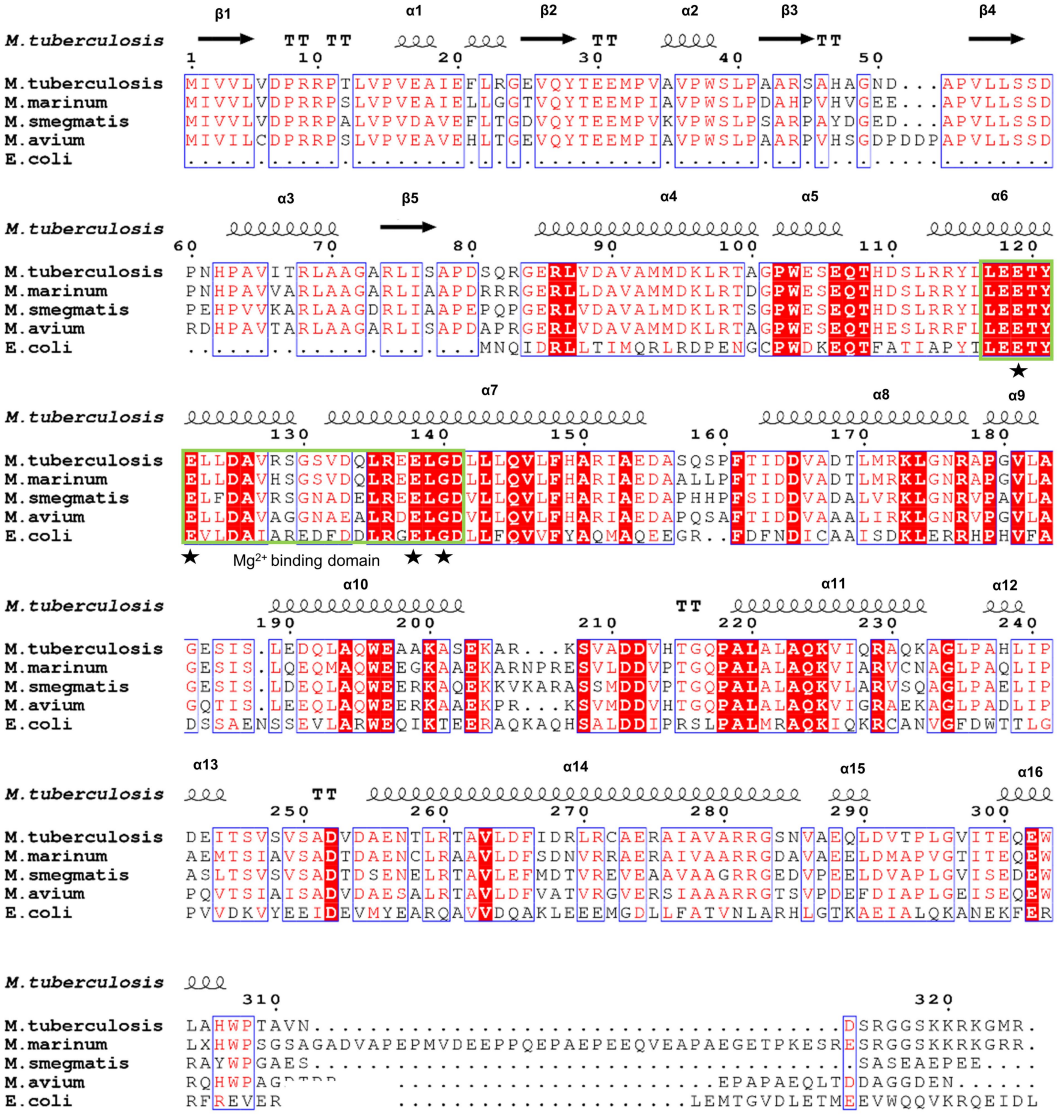
Sequence alignment of Mtb-MazG with its orthologs. Sequence alignment was performed using the ClustalX and ESpript v.3.0 programs. Identical and similar residues are shown in white text on a red background and in red text on a white background, respectively. The magnesium binding domain was highlighted with a green box. The star denotes residues that are bound with magnesium.

**Figure 6:**
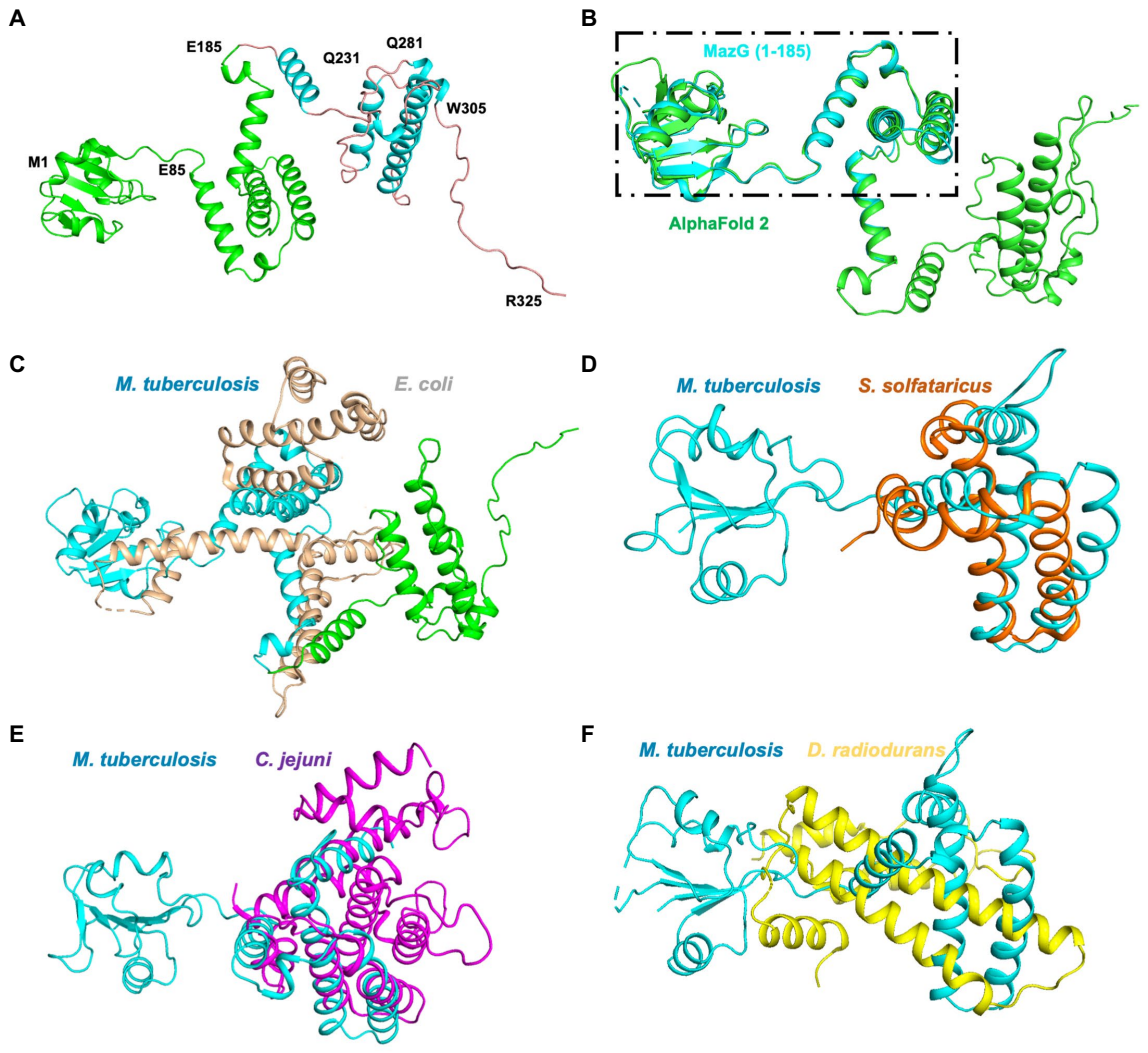
Structure superimposition of Mtb-MazG and its homologous proteins. **(A)** The AlphaFold2-predicted structure of MazG is shown in the cartoon model with different colors. The crystal structure of MazG (1–185) is shown in green color. Different numbers denote the truncated fragments. **(B)** The structural superposition of Mtb MazG (1–185; cyan) and AlphaFold2-predicted full-length MazG (green). **(C)** The AlphaFold2-predicted full-length MazG (cyan and green) was superimposed with *E. coli* MazG (PDB code 3CRC; wheat). **(D–F)** Structural superimposition of Mtb MazG (1–185; cyan) with *S. solfataricus* MazG (PDB code 1VMG; orange), *C*. *jejuni* MazG (PDB code 1W2Y; purple), and *D. radiodurans* MazG (PDB code 2YFD; yellow).

To further identify the critical configuration elements of MazG in cell metabolism, we performed a structural-based alignment for Mtb-MazG with its different bacteria orthologs with Clustal X software (Figure 5). The results showed that the NTD and CTD of MazG are conserved in different bacteria, including *M. tuberculosis*, *M. marinum, M. smegmatis, M. avium,* and *E. coli*. However, the mycobaterial MazGs possess one specific N-terminal extra region, while *E. coli* MazG does not (Figures 2A,B, 5). When superimposing the crystal structure of Mtb-MazG with the AlphaFold2-predicted model, the RMSD value was only 1.207 Å, indicating the two structures were very similar (Figures 6A,B). Also, Mtb-MazG showed different overall folds with homologs from different species. Although the amino acid sequence showed high similarity (~39%) between *E. coli* MazG (*Ec*MazG) and Mtb-MazG, the overall structure was dramatically different, evidenced by that the RMSD value was 32.275 Å when superimposing the Mtb-MazG structure with the *Ec*MazG (PDB ID: 3CRC; Figure 6C; Lee et al., 2008). Interestingly, MazG proteins from different mycobacterial species might have conserved structures, as superimposing the Alphafold-predicative MazG models of the *M. marinum*, the *M. avium*, and the *M. smegmatis* with Mtb-MazG showed that the RMSD values were 1.723 Å, 0.670 Å, and 0.500 Å, respectively. Moreover, superimposing the *C. jejun* dUTPase structure (PDB ID: 1W2Y; Moroz et al., 2004), the *S. solfataricus* MazG (PDB ID: 1VMG), and the *D. radiodurans* MazG (PDB ID: 2YFD; Goncalves et al., 2011) with Mtb-MazG showed that the RMSD values were 2.165 Å, 5.332 Å, and 13.52 Å, respectively (Figures 6D,F).

## Discussion

MazG belongs to the all-α NTP pyrophosphatases (Galperin et al., 2006), which exist from viruses and bacteria to humans (Song et al., 2015; Rihtman et al., 2019; Zaide et al., 2020; Huang et al., 2021; Wood et al., 2021; Han et al., 2022). As a housecleaning enzyme, MazG functions at the cell nucleotide metabolism by degrading the non-canonical NTPs, preventing mutagenesis and DNA damage. Moreover, MazG helps bacteria in response to oxidative stress in *E. coli* (Han and Eiteman, 2018), *B. anthracis* (Zaide et al., 2020), and *Mycobacterium* (Shi et al., 2019). In addition, many studies show that MazG is critical in regulating the DNA damage response in mycobacterium (Lu et al., 2010; Lyu et al., 2013; Fan et al., 2018; Shi et al., 2019). Deletion of *MazG* in mycobacteria resulted in a 20-fold increase in the frequency of genomic CG-TA mutation both under oxidative stress and the stationary phase of growth (Fan et al., 2018). This suggests that MazG plays an important function in *Mycobacterium tuberculosis* infection.

In the current study, we solved the crystal structure of Mtb-MazG (1–185), which shows distinct overall architecture with other all-α NTP pyrophosphatases. The specific NE domain of Mtb-MazG comprises α-helices and β-stands (Figure 2), which is indispensable for its enzymatic activity (Figure 4). We found that the 1–85 sequence deletion significantly impacts MazG enzyme activity. The formation of dimeric MazG is related to multiple amino acid sites (Figure 2D). Therefore, we speculated that the deletion of the 1–85 sequence might have a negative impact on MazG dimeric formation, thus affecting the overall enzyme activity. The NTD domain is composed of α-helices and has a typical EEXX (E/D) motif, which forms the magnesium ion binding sites with four residues Glu119, Glu122, Glu138, and Asp141 (Figure 4). The four potential active site residues, the EEXX(E/D) motif, are frequently found in different enzymes, requiring magnesium or manganese ions for their activities (Peters and Croteau, 2002; Lee et al., 2008). Also, *Campylobacter jejuni* dUTPase coordinates a magnesium ion with acidic Glu46, Glu49, Glu74, and Asp77, and hydrolyzes the substrate using basic residues of Lys175, Arg182, and Lys194 (Moroz et al., 2004). The active site of *Ec*MazG contains six conserved acidic residues (Glu171, Glu172, Glu175, Glu192, Glu193, and Asp196). However, only three (Glu172, Glu193, and Asp196) are involved in the magnesium coordination for NTPase activity. The NTD and CTD of Mtb-MazG show a high similarity with other bacterial homologs in amino acid sequences. All of them are composed of α-helices that play essential roles for (d)NTPs hydrolysis. However, the overall structures are quite different (Figure 6). Thus, the specific 3D structure of Mtb-MazG may contribute to the antibacterial lethality and coordinate the metabolic adaption of dormant Mtb. Further studies targeting MazG could benefit us in identifying potential compounds in the treatment of TB.

## Materials and methods

### Protein expression and purification

The gene encoding Mtb-MazG (NP_215537) of *M.tuberculosis* H37Rv was synthesized and constructed into the pSMT3 vector to produce the N-terminal 6× His-SUMO tagged fusion protein. The expression and purification processes were similar to previous works (Chen et al., 2020; Gao et al., 2021). In brief, Mtb*-*MazG was expressed in *Escherichia coli* BL21(DE3) cells. Cell cultures in LB medium were induced with 0.5 mM isopropyl-β-D-thiogalactoside (IPTG) at 16°C for 20 h when OD_600_ reached 0.6. Then, cells were suspended in buffer A (50 mM Tris–HCl, pH 8.0, 300 mM NaCl, 5% glycerol, 10 mM Imidazole) and lysed by a high-pressure homogenizer. Mtb*-*MazG was then purified by Ni-NTA affinity chromatography, and the His-sumo tag was removed by ULP1 enzyme cleavage, followed by an additional Ni-NTA affinity chromatography. The target protein was then applied to a Superdex 200 16/600 gel filtration column pre-equilibrated in buffer B (20 mM Tris–HCl, pH 8.0, 150 mM NaCl, 2 mM DTT). Finally, 10 ~ 20 mg per liter LB medium of the target protein with purity above 95% was obtained.

Site-directed mutants were constructed according to the standard QuikChange Site-Directed Mutagenesis protocol (Stratagene, United States) using the wild-type (WT) Mtb*-*MazG as the template. All the constructs were confirmed by DNA sequencing. The expression and purification of truncations and mutants were the same with the WT Mtb*-*MazG.

### Limited proteolysis

The full-length MazG was incubated with different proteases (Hampton Research, Proti-Ace • Proti-Ace 2, HR2-432) at 37°C for 2 h in the reaction buffer (20 mM Tris–HCl, pH 8.0, 150 mM NaCl, 5 mM MgCl_2_). The reaction was stopped by heating at 95°C for 10 min. The ratio of enzyme to protein is 1:1000 (mol: mol).

### Dynamic light scattering measurement

The Dynamic light scattering (DLS) data were collected on the DYNAMICS software from DynaPro NanoStar (Wyatt Technology), operating at a light source wavelength of 658 nm and a fixed scattering angle of 90°. The fresh proteins were diluted to 1 mg/mL with a buffer containing 20 mM Tris–HCl (pH 8.0), 150 mM NaCl, 2 mM DTT at 25°C.

### Crystallization and data collection

Mtb*-*MazG (1–185) protein (~20 mg/mL) in a buffer with 20 mM Tris pH 8.0, 150 mM NaCl, 2 mM DTT, and 5 mM MgCl_2_ was crystallized at 18°C using the vapor-diffusion method by mixing with equal volume reservoir solution (0.1 M HEPES pH 7.5, 10% W/V PEG8000, 8% ethylene glycol). Crystals grew out at 18°C after 3 days. Diffraction data were collected with crystals flash-frozen in the crystallization buffer supplemented with 20% (v/v) glycerol. Integration, scaling, and merging of the diffraction data were performed using the HKL2000 suite.

### Structure determination and refinement

The Mtb*-*MazG structure was determined by the molecular replacement method using the structure (ID: AF-P96379-F1) predicted by AlphaFold2 as the search model (Tunyasuvunakool et al., 2021). Crystal structure refinements were performed with the program PHENIX (Adams et al., 2010). COOT and PyMOL software were used for model building and analysis (Emsley et al., 2010). The collected data and refinement statistics are summarized in Table 1.

**Table 1:** Data collection and refinement statistics.

Items	MazG (1–185)
PDB code	7YH5
Data collection
Space group	P4212
Cell dimensions	*a* = 109.31 Å, *b* = 109.31 Å, *c* = 242.91 Å
	*α* = 90°, *β* = 90°, *γ* = 90°
Resolution (Å)^a^	35.86–2.70 (2.796–2.70)
R _merge_ (%)^b^	1.654 (35.19)
/	17.90 (1.88)
CC_1/2_	1 (0.729)
Wilson B-factor	87.7
Completeness (%)	99.85 (99.98)
Refinement
No. reflections	41,349 (4072)
R_work_ (%)^c^	25.80 (32.82)
R_free_ (%)^d^	29.62 (38.06)
Macromolecules	7,077
Protein	984
Ligand	6
Water	35
RMSD
Bond lengths (Å)	0.010
Bond angles (°)	1.01
Ramachandran analysis (%)
Favored	95.93
Allowed	4.07

^a^Values in parentheses are for the highest resolution shell. ^b^R_merge_ = Σ|I_i_ − <I > |/Σ|I|, where Ii is the intensity of an individual reflection and is the average intensity of that reflection. ^c^R_work_ = Σ||F_o_| − |F_c_||/Σ|F_o_|, where F_o_ and F_c_ are the observed and calculated structure factors for reflections, respectively. ^d^R_free_ was calculated as R_work_ using the 5% of reflections that were selected randomly and omitted from refinement.

### Structure-based sequence alignment

Multiple alignments of amino acid sequences of different MazG proteins were performed using ClustalX v.2 program. Secondary structure alignment was generated by DSSP v.2.0 and ESpript v.3.0.^1^

### Small-angle X-ray scattering analysis

The MazG Small-angle X-ray scattering (SAXS) data were collected at beamline BL19U2 of the Shanghai Synchrotron Radiation Facility with a radiation wavelength of 1.03 Å. The protein samples were prepared at concentrations of 1 mg/mL in 20 mM Tris–HCl (pH 8.0), 150 mM NaCl. Each blank or sample was measured in triplicate, and the sample measurements were adjusted by subtracting the scattering from the buffer alone. SAXS data analysis program ATSAS 2.84 suite was used to process and analyze the MazG scattering curve (Manalastas-Cantos et al., 2021). The radius of gyration (Rg) of globular, rod shape (Rc), and flat shape (Rf) were determined by applying the Guinier approximation equation in primusqt. GNOM software coupled with ATSAS was used for the evaluation of the maximum particle dimension (Dmax) and the distance distribution function plot (P(R)). The SAXSMow program was used to determine the molecular mass of MazG. DAMMIF program was used to construct 10 independent *ab initio* models. The software PyMol was used to show the model shape.

### Enzymatic activity measurement

The NTP-PPase activity of Mtb*-*MazG was assayed by measuring the hydrolyzed product, PPi, by an enzyme-coupled colorimetric assay (Molecular Probes) with a detection limit of 0.2 μM. The standard NTP-PPase assay was carried out in a 20 μl reaction buffer (20 mM Tris–HCl, pH 8.0, 150 mM NaCl, 5 mM MgCl_2_) containing 1 μg of Mtb*-*MazG and an appropriate amount of nucleoside triphosphates at 37°C for 10 min. The reaction was stopped by heating at 70°C for 10 min, and 5 μl of the reaction product was applied for the PiPer pyrophosphate assay (Molecular Probes) according to the manufacturer’s instructions. The reaction without Mtb*-*MazG or substrates was carried out as a background control.

### Circular dichroism spectrometry

The Circular dichroism (CD) spectra were measured on a Chirascan Plus spectropolarimeter in the far-ultraviolet region (260 nm-190 nm) in a step of 1 nm. Records on protein solutions (0.2 mg/mL in PBS) employing a cell with a path length of 1 mm at 18°C were obtained. Each spectrum reported is an average of three scans.

### Statistical analysis

Each experiment was performed at least three times. All experiment data were analyzed using GraphPad Prism 7.0 (GraphPad software Inc. United States) and were presented as mean values ± SD. Statistical analysis was performed using a t-test (*: *p* < 0.05; **: *p* < 0.01; ***: *p* < 0.001).

## Data availability statement

The datasets presented in this study can be found in online repositories. The names of the repository/repositories and accession number(s) can be found in the article/Supplementary material.

## Author contributions

JL and GZ conceived and supervised the study. SeW, BG, AC, ZZ, ShW, and LL performed experiments and data analysis. JL and SeW wrote the manuscript. All authors contributed to the article and approved the submitted version.

## Funding

This work was supported by grants from the National Key Research and Development Project of China (2021YFC2301500 and 2016YFA0500600), the National Natural Science Foundation of China (32161160323), and the Shanghai Committee of Science and Technology (20XD1400800).

## Conflict of interest

ShW was employed by Shanghai Zelixir Biotech Company Ltd.

The remaining authors declare that the research was conducted in the absence of any commercial or financial relationships that could be construed as a potential conflict of interest.

## Publisher’s note

All claims expressed in this article are solely those of the authors and do not necessarily represent those of their affiliated organizations, or those of the publisher, the editors and the reviewers. Any product that may be evaluated in this article, or claim that may be made by its manufacturer, is not guaranteed or endorsed by the publisher.

## Footnotes

^1^ http://espript.ibcp.fr/ESPript/ESPript/
