# Supplementary material for: Structural analysis of the housecleaning nucleoside triphosphate pyrophosphohydrolase MazG from Mycobacterium tuberculosis
Source: Front Microbiol. 2023 Mar 1;14:1137279. doi: 10.3389/fmicb.2023.1137279 (PMC10014863; doi:10.3389/fmicb.2023.1137279)
Supplement: Supplementary file 1 [file Data_Sheet_1.docx]

**Structural analysis of the housecleaning nucleoside triphosphate pyrophosphohydrolase MazG** **from** ***Mycobacterium tuberculosis***

Sen Wang^1^, Baocai Gao^1^, Anke Chen^1^, Zhifei Zhang^1^, Sheng Wang^2^, Liangdong Lv^3^, Guoping Zhao^1,4,*^, Jixi Li^1,5,*^

^1^State Key Laboratory of Genetic Engineering, School of Life Sciences and Huashan Hospital, MOE Engineering Research Center of Gene Technology, Shanghai Engineering Research Center of Industrial Microorganisms, Fudan University, Shanghai 200438, China

^2^Shanghai Zelixir Biotech Company Ltd., Shanghai 200030, China

^3^School of Basic Medical Sciences, Fudan University, Shanghai 200032, China

^4^Key Laboratory of Synthetic Biology, CAS Center for Excellence in Molecular Plant Sciences, Shanghai Institute of Plant Physiology and Ecology, Chinese Academy of Sciences, Shanghai 200032, China

^5^Shanghai Key Laboratory of Infectious Diseases and Biosafety Emergency Response, National Medical Center for Infectious Diseases, Huashan Hospital, Fudan University, Shanghai, 200040, China

^*^To whom correspondence should be addressed. Email: [lijixi@fudan.edu.cn](mailto:lijixi@fudan.edu.cn) or [gpzhao@sibs.ac.cn](mailto:gpzhao@sibs.ac.cn)

**Supplementary Materials**


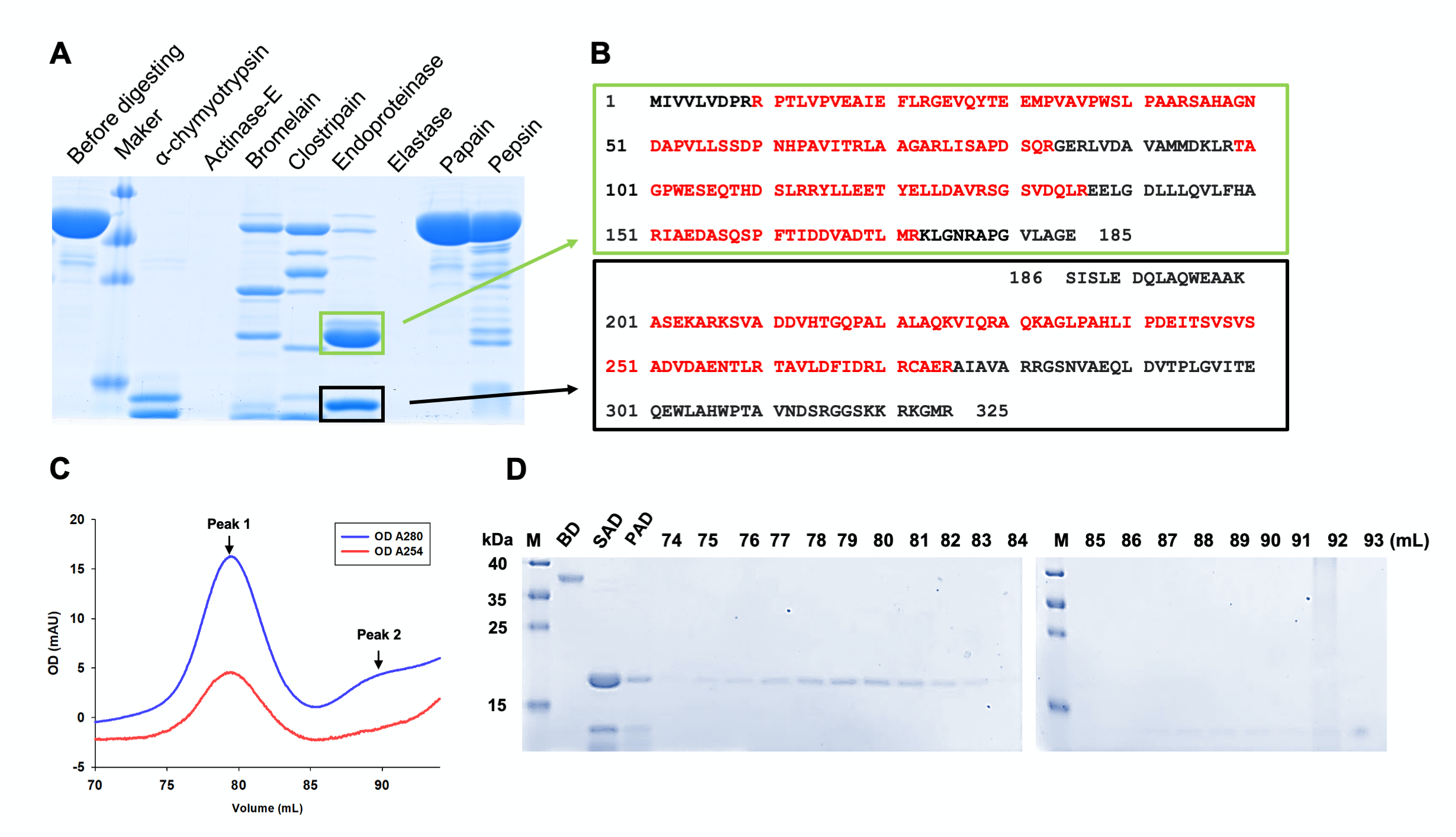


**Figure S1. The limited proteolysis and mass spectrometry results of Mtb-MazG.**

**A.** The SDS-PAGE result of Mtb-MazG using different proteases. **B.** The mass spectrometry (MS) result of MazG with two bands cleaved by endoproteinase. The green and black boxes denote for the N-terminal region and C-terminal region, respectively. The peptide sequences identified by MS were highlighted with red color. **C-D.** The gel filtration and SDS-PAGE result of full-length MazG after in-situ endoproteinase digestion. M: protein markers. BD: before digestion; SAD: supernatant after digestion; PAD: precipitate after digestion.
